# Acute Pericarditis as a Presentation of Adrenal Insufficiency

**DOI:** 10.7759/cureus.2474

**Published:** 2018-04-13

**Authors:** Sukesh Manthri, Sindhura Bandaru, Abdisamad Ibrahim, Chaitanya K Mamillapalli

**Affiliations:** 1 Department of Geriatrics, Saint Louis University School of Medicine; 2 Department of Internal Medicine, Southern Illinois University School of Medicine; 3 Department of Endocrinology, Springfield Clinic, Springfield, USA

**Keywords:** secondary adrenal insufficiency, acute pericarditis, cardiac tamponade, adrenal insufficiency, isolated acth deficiency, acute adrenal insufficiency

## Abstract

Acute pericarditis as a presenting sign of adrenal insufficiency is rarely reported. We present a rare case that highlights pericarditis as a clinical presentation of secondary adrenal insufficiency later complicated by cardiac tamponade. A 44-year-old lady who presented to the hospital with a one-day history of pleuritic chest pain and shortness of breath. In the emergency room, she had a blood pressure of 70/35 mmHg. Laboratory evaluation revealed white blood cell count of 16.08 k/cumm with neutrophilia, normal renal function and elevated troponin (0.321 ng/mL, normal 0.000-0.028). An electrocardiogram (EKG) showed sinus tachycardia, low voltage, PR suppression and ST changes consistent with acute pericarditis. Echocardiogram showed small pericardial effusion without tamponade physiology. Infectious workup was negative; she was thought to have acute adrenal insufficiency likely secondary to viral pericarditis. We treated the patient with high dose nonsteroidal anti-inflammatory drugs (NSAIDS) and hydrocortisone. Three weeks later, she presented to emergency room with complaints of persistent nausea, vomiting, chills, weakness. Her blood pressure was 49/23 mmHg. Random serum cortisol level was <1.2 mcg/dl (normal A.M. specimens 3.7-19.4 mcg/dl). Echocardiogram showed loculated pericardial fluid adjacent to the right ventricle with echocardiographic evidence of tamponade. Emergent pericardiocentesis yielded 250 ml of straw color fluid. Blood pressure improved after the procedure. The patient was initially started on IV stress dose steroids, but following clinical stabilization, hydrocortisone was switched to a physiological dose of 15 mg in am and 10 mg in pm. Although the mechanism of pericarditis in adrenal failure is unknown, this clinical presentation may help early diagnosis of adrenal failure and pericarditis. Early recognition and prompt treatment of this rare presentation are critical to prevent morbidity and mortality.

## Introduction

Acute pericarditis as a presenting sign of adrenal insufficiency is rarely reported. Isolated adrenocorticotropic hormone (ACTH) deficiency as a cause of secondary adrenal insufficiency is a rare clinical condition with varied clinical presentation and occasionally fatal course. We present a rare case that highlights pericarditis as a clinical presentation of isolated ACTH deficiency later complicated by cardiac tamponade.

## Case presentation

**Figure 1:**
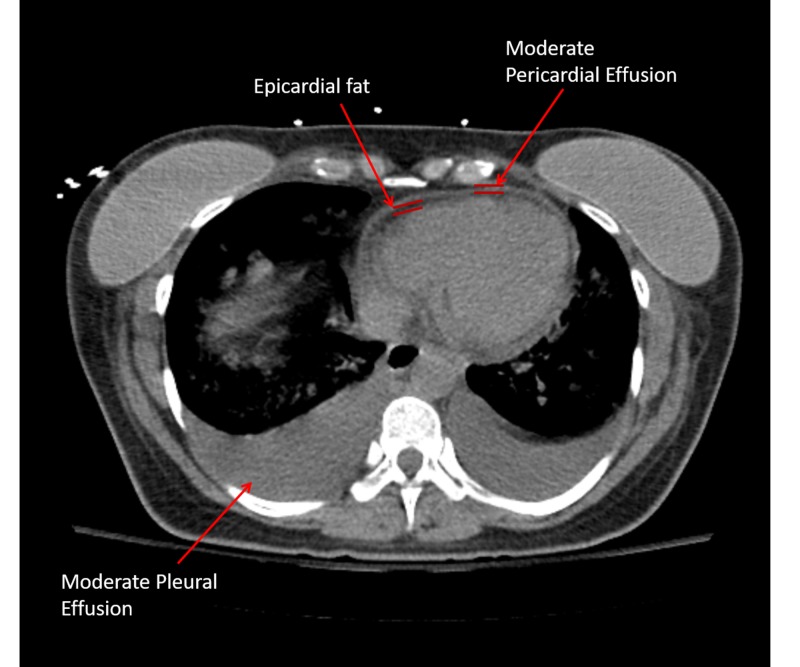
Computed tomography (CT) chest showing pericardial effusion. Computed tomography demonstrated a moderate pericardial effusion in a patient with a normal heart size, moderate pleural effusions and marked peribronchovascular ground glass opacifications (representative of pulmonary edema).

A 44-year-old female with no significant past medical history presented with a one-day history of pleuritic chest pain and dyspnea. She denied any history of fever, chills, nausea, and vomiting. In the emergency room, she had a temperature of 35.1°C, heart rate of 107 per minute, respiratory rate of 22 per minute, and blood pressure of 70/35 mmHg; she was alert but lethargic, and no focal neurological deficit was noted. Laboratory evaluation revealed hemoglobin of 12.7 gm/dl, white blood cell (WBC) count of 16.08 k/cumm with neutrophilia, normal renal function and elevated troponin (0.321 ng/ml, normal 0.000-0.028). An electrocardiogram (EKG) showed sinus tachycardia, low voltage, PR suppression and ST changes consistent with acute pericarditis. Echocardiogram showed normal left ventricular systolic function with calculated ejection fraction of 70%, small pericardial effusion without tamponade physiology. Computed tomography (CT) (Figure 1) demonstrated a moderate pericardial effusion in a patient with a normal heart size, moderate pleural effusions and marked peribronchovascular ground glass opacifications (representative of pulmonary edema).

Her hypotension persisted despite aggressive fluid resuscitation, and she was started on vasopressors norepinephrine and epinephrine to maintain mean arterial pressure >65. Blood cultures were ordered, and she was started on broad-spectrum antibiotics consisting of vancomycin 1.5 gm IV every eight hours (~15 mg/kg), piperacillin-tazobactam 4.5 gm IV every eight hours and levofloxacin 750 mg IV once daily. Further evaluation revealed C-Reactive protein of 13.8 mg/dl (normal <0.6 mg/dl), random serum cortisol level of <1.0 mcg/dl collected at 09:00 AM (normal A.M. specimens 3.7-19.4 mcg/dl). She was diagnosed with acute pericarditis and was started on Ibuprofen 800 mg every eight hours along with hydrocortisone 100 mg IV every eight hours for adrenal insufficiency. Patient clinically improved after starting steroids and vasopressors were weaned. As her infectious workup was negative, antibiotics were stopped. She was thought to have stress-induced relative adrenal insufficiency likely secondary to viral pericarditis; she was discharged home after 11 days, with a tapering dose of hydrocortisone over four days and was advised to closely follow up with primary care physician and cardiologist.

Three weeks later, she presented to local emergency room with complaints of persistent nausea, vomiting, chills, and weakness. In the emergency room, she had a temperature of 33.1°C (91.5°F), heart rate of 103 per minute, respiratory rate of 25 per minute, and blood pressure of 49/23 mmHg; she was alert but lethargic, and no focal neurological deficit was noted. Laboratory evaluation revealed hemoglobin of 11.9 gm/dl, WBC count of 23.37 k/cumm with neutrophilia. Comprehensive metabolic panel showed hyponatremia (130 mmol/L, 135-147), hypokalemia (3.4 mol/L, 3.5-5.0), and elevated creatinine of 2.9 mg/dl (normal 0.6-1.1 mg/dl). Further workup revealed elevated troponin (0.165 ng/mL, normal 0.000-0.028) and low 8:00 AM serum cortisol level of <1.2 mcg/dl (normal A.M. specimens 3.7-19.4 mcg/dl). EKG showed sinus rhythm with frequent supraventricular premature complexes, low QRS voltage and nonspecific ST and T wave changes. Her hypotension persisted despite aggressive fluid resuscitation, and she was started on vasopressors norepinephrine and dopamine to maintain mean arterial pressure >65. Echocardiogram showed loculated pericardial fluid adjacent to the right ventricle with echocardiographic evidence of tamponade. Emergent pericardiocentesis yielded 250 ml of straw color fluid. Blood pressure improved after the procedure.

The pericardial fluid contained 3.287 k/cumm WBC mainly polymorphonuclears but negative cultures (aerobic, anaerobic, acid-fast bacilli smear and fungal cultures). After the urgent pericardiocentesis, she was still noted to have a small effusion and ultimately pericardial drain was placed. Unfortunately, her hospital course was complicated by left-sided pneumothorax with thoracostomy tube placement. Repeat echocardiogram showed normal right ventricular size, normal systolic function with resolved pericardial effusion seen earlier. Endocrinology was consulted for further workup and management of adrenal insufficiency of unclear etiology. Table 1 below shows results of the endocrine evaluation, with notable findings of mildly elevated thyroid-stimulating hormone (TSH) at 6.1 mcIU/ml with normal free T4 likely suggesting sick euthyroid syndrome.

**Table 1:** Results of endocrine studies. ACTH: Adrenocorticotropic hormone; LH: Luteinizing hormone; FSH: Follicle-stimulating hormone; TSH: Thyroid-stimulating hormone; T4: Thyroxine.

Endocrine studies	Result	Reference range
Random serum cortisol	1.2 mcg/dl	3.7-19.4 mcg/dl (A.M. specimens)
ACTH	15 pg/ml (done after steroid administration)	10 to 60 pg/ml (A.M. specimens)
LH	1.1 mIU/ml	Follic phase: 1.80 to 11.78 mIU/ml; Mid cycle: 7.59 to 89.08 mIU/ml; Luteal phase: 0.56 to 14.00 mIU/ml; Post menopausal W/O HRT: 5.16 to 61.99 mIU/ml
FSH	7.7 mIU/ml	Follic phase: 3.03 to 8.08 mIU/ml; Mid cycle: 2.55 to 16.69 mIU/ml; Luteal phase: 1.38 to 5.47 mIU/ml; Post menopausal: 26.72 to 133.41 mIU/ml
Ultrasensitive TSH	6.10 mcIU/ml	0.35-4.94 mcIU/ml
Free T4	1.0 ng/dl	0.9-1.5 ng/dl

Rheumatology was consulted for further evaluation of pericarditis, and further workup was negative for Lyme disease antibody, rheumatoid factor, antinuclear antibodies (ANA), Immunoglobulin G (IgG) antibody, smith antibody, parvovirus B19 IgG and Immunoglobulin M (IgM) antibody, human immunodeficiency virus (HIV), and compliments levels were normal. Interferon-gamma release assay (TB Gold) testing was negative. The patient was initially started on IV stress dose steroids, but following clinical stabilization, hydrocortisone was switched to a physiological dose of 15 mg in AM and 10 mg in PM. She was discharged with advice for follow-up with an endocrinologist in one month.

Upon follow-up at one month, she has done well as a whole except for complaint of increased appetite and weight gain of 20 lbs since her hospitalization. Unfortunately, ACTH level was not done before the commencement of steroids during her admission, complicating the differentiation of primary and secondary adrenal insufficiency. She did not need mineralocorticoid replacement, therefore, she was diagnosed with secondary adrenal insufficiency rather than primary adrenal insufficiency. Pituitary magnetic resonance imaging (MRI) showed mildly heterogeneous enhancement within a normal-sized pituitary gland, no discrete mass was identified. Due to significant weight gain, the dose of hydrocortisone was reduced from 22.5 mg to 20 mg PO daily (15 mg in AM and 5 mg in PM). Unfortunately, the patient was lost to follow-up.

## Discussion

Secondary adrenal insufficiency can be caused by diseases that interfere with corticotropin (ACTH) secretion by the pituitary gland. Most common cause of secondary adrenal insufficiency is discontinuation of prolonged steroid treatment. Our patient did not have exposure to steroids before the current presentation. Secondary adrenal insufficiency can be a component of panhypopituitarism resulting from pituitary destruction secondary to mass lesions like macroadenomas, craniopharyngiomas, infectious diseases such as histoplasmosis or tuberculosis, infiltrative disorders, lymphocytic hypophysitis, head trauma, and large intracranial artery aneurysm.

Isolated ACTH deficiency occurs in patients with lymphocytic hypophysitis, Sheehan’s syndrome, radiotherapy, traumatic brain injury and subarachnoid hemorrhage. Isolated ACTH deficiency (IIAD) syndrome is a heterogeneous, rare disorder with nonspecific clinical symptoms and variable presentations and is a diagnosis of exclusion. In a large case series published by Hannon et al., the following diagnostic criteria were used to define IIAD [1]:

1. Evidence of low cortisol, with abnormal cosyntropin test, associated with inappropriately low ACTH level.

2. An absence of other pituitary hormone deficits.

3. Normal pituitary imaging.

4. Intact renin/aldosterone axis.

5. No history of brain trauma or clinical features of lymphocytic hypophysitis.

Unlike primary adrenal insufficiency, mineralocorticoid axis is preserved in patients with secondary adrenal insufficiency. Although we do not have pretreatment ACTH level, our patient did not require mineralocorticoid replacement, which is in favor of secondary adrenal insufficiency. The patient had regular menses before the hospital presentation indicating intact gonadotropic axis, and thyroid function tests were not suggestive of secondary hypothyroidism. There was no imaging evidence of pituitary abnormalities; these findings were supportive of idiopathic isolated ACTH deficiency as the cause of secondary adrenal insufficiency in our patient.

Acute pericarditis as a suspected symptom of adrenal failure has rarely been reported. Table 2 summarizes published cases with cardiac manifestations of secondary adrenal insufficiency [2]. Mineralocorticoid deficiency leads to hyponatremia, hyperkalemia and metabolic acidosis [3]. The glucocorticoids maintain inotropy and modulate vascular response to the beta agonists. Lack of glucocorticoids manifests as hypotension characterized by tachycardia, reduced stroke volume and decreased peripheral vascular resistance, as well as hyponatremia secondary to ADH-medicated water retention.

**Table 2:** Published cases of cardiac manifestations of secondary adrenal insufficiency. ACTH: Adrenocorticotropic hormone; CHF: Congestive heart failure; CMP: Cardiomyopathy; EF: Ejection fraction.

Study	Patient age (years)	Etiology of adrenal insufficiency	Cardiac manifestation	Outcome
Eto K et al. [4]	62 M	Empty sella	CHF, CMP, QT prolongation	Resolved with replacement
Bao SS et al. [5]	35 F	Sheehan syndrome	Dilated CMP, CHF	Resolved with replacement
Ukita C et al. [6]	69 F	ACTH deficiency	Takotsubo, CMP	Resolved with replacement
Gotyo N et al. [7]	70 M	Idiopathic ACTH deficiency	Takotsubo, CMP, Torsade de Pointes	Resolved with replacement
Giraldi et al. [8]	60 M	Idiopathic ACTH deficiency	Pericardial effusion	Resolved with replacement

The presentation of acute adrenal insufficiency usually includes nausea, vomiting, severe hypotension, and in severe cases with hypovolemic shock. Patients with acute cardiac tamponade may present with similar presentation with features of hypoperfusion, and in the absence of high clinical suspicion diagnosis of adrenal insufficiency can be missed with potentially fatal outcomes.

Acute pericardial effusion is mainly idiopathic in developed countries, with no identification of any specific cause and presumed to be of viral origin. Tucker et al. [9] reported the clinical range of serositis with autoimmune endocrinopathy in their 20 patients. The occurrence of pericarditis with tamponade as an initial manifestation of Addison’s disease extends the clinical spectrum. However, three of whom were already known to have idiopathic primary hypoadrenalism by the time they developed pericardial tamponade. The fact that pericarditis antedated proven endocrinopathy is in keeping with the observation that endocrinopathy-related serositis can also occur after asymptomatic intervals of months or years, even in patients already receiving replacement therapy, perhaps since its etiopathogenesis involves not only endocrine failure but also derangements in immune complex and immunogenetic status [10].

Torfoss et al. [11] reported two cases who presented with cardiac tamponade and developed clinical adrenal insufficiency within a few weeks and speculated whether there could be a common etiological factor for the pericarditis and the triggering of the adrenal destruction. Taxter et al. [12] reported a pediatric case with pericarditis in association with hypocortisolism from a nonautoimmune cause and proposed that hypocortisolism itself may lead to pericarditis in some patients. Extensive workup for the autoimmune process was negative in our patient.

The etiopathogenesis of idiopathic recurrent acute pericarditis remains controversial and can be associated with infectious, autoimmune and autoinflammatory pathways. Echo-virus and Coxsackie are the most frequently involved viruses, Mycobacterium tuberculosis and Coxiella burnetii are the most common bacteria, but in 85% of cases, it remains "idiopathic." The recurrence of pericarditis is not rare; it occurs in 20 to 50% of cases of pericarditis and requires administration of immunosuppressive drugs [13]. Kawahara et al. [14] published a case report of tuberculous Addison disease with recurrent nontuberculous pericarditis. There is a possibility that infection may have induced pericarditis with pericardial effusion and leading to adrenal insufficiency in our patient. However, negative infectious workup including cultures along with quick treatment response to stress doses of hydrocortisone ruled out this possibility.

## Conclusions

In conclusion, isolated ACTH deficiency is a rare clinical condition with a heterogeneous clinical presentation. To the best of our knowledge, our case report appears to be only the second reported instance of a patient with isolated ACTH deficiency presenting with a pericardial effusion. Early recognition and prompt treatment of this unusual presentation are critical to avoid morbidity and mortality.
